# When the human viral infectome and diseasome networks collide: towards a systems biology platform for the aetiology of human diseases

**DOI:** 10.1186/1752-0509-5-13

**Published:** 2011-01-21

**Authors:** Vincent Navratil, Benoit de Chassey, Chantal Rabourdin Combe, Vincent Lotteau

**Affiliations:** 1 Université de Lyon, IFR128 BioSciences Lyon-Gerland, Lyon 69007, France; 2 INRA, UMR754, rétrovirus et pathologie comparée, Lyon 69007, France; 3 Inserm Unit 851, Lyon 69007, France; 4 Hospices Civils de Lyon, Hôpital de la Croix-Rousse, Laboratoire de virologie, Lyon 69004, France; 5 Pôle Rhône Alpes de Bioinformatique, Université Lyon 1, Batiment Gregor Mendel, 16 rue Raphaël Dubois, 69622 Villeurbanne cedex, France

## Abstract

**Background:**

Comprehensive understanding of molecular mechanisms underlying viral infection is a major challenge towards the discovery of new antiviral drugs and susceptibility factors of human diseases. New advances in the field are expected from systems-level modelling and integration of the incessant torrent of high-throughput "-omics" data.

**Results:**

Here, we describe the Human Infectome protein interaction Network, a novel systems virology model of a virtual virus-infected human cell concerning 110 viruses. This *in silico *model was applied to comprehensively explore the molecular relationships between viruses and their associated diseases. This was done by merging virus-host and host-host physical protein-protein interactomes with the set of genes essential for viral replication and involved in human genetic diseases. This systems-level approach provides strong evidence that viral proteomes target a wide range of functional and inter-connected modules of proteins as well as highly central and bridging proteins within the human interactome. The high centrality of targeted proteins was correlated to their essentiality for viruses' lifecycle, using functional genomic RNAi data. A stealth-attack of viruses on proteins bridging cellular functions was demonstrated by simulation of cellular network perturbations, a property that could be essential in the molecular aetiology of some human diseases. Networking the Human Infectome and Diseasome unravels the connectivity of viruses to a wide range of diseases and profiled molecular basis of Hepatitis C Virus-induced diseases as well as 38 new candidate genetic predisposition factors involved in type 1 *diabetes mellitus*.

**Conclusions:**

The Human Infectome and Diseasome Networks described here provide a unique gateway towards the comprehensive modelling and analysis of the systems level properties associated to viral infection as well as candidate genes potentially involved in the molecular aetiology of human diseases.

## Background

Infectious diseases cause million of deaths as reported by the World Health Organization (WHO). In this context, emerging and re-emerging infectious diseases - such as flu, AIDS, tuberculosis, hepatitis - stress the need to accelerate and rationalize the development of better diagnostics, treatments and preventions. It is widely accepted that appearance and severity of infectious diseases depend on the ability of viruses to interfere with host cell functions and defence. Indeed, during virus-host co-evolution, hosts have selected an arsenal of complex defence mechanisms to eliminate viruses from the organism. Conversely, because of their high replication and propagation rate, viruses have evolved strategies, at least in part driven by molecular interactions, to circumvent host cellular defence and to maintain their control on the cellular machinery. Comprehensive understanding of how viruses and their host interact appears as a major milestone in the quest of deciphering viral infection processes and the molecular aetiology of some complex diseases.

Owing to new advances in high-throughput profiling of an infection, *i.e. *infectomics, it is now possible to investigate viral infection at the systems level. The virus-host interactome, *i.e. *the whole set of protein interactions occurring within virus-infected cells, has been investigated in the last decades by the mean of low scale experiments focusing on single viral proteins. Recently, interactomics relying on high-throughput technologies (yeast two-hybrid, co-immunoprecipitation coupled to mass spectrometry) have boosted virus-host systems biology [1,2]. Improved procedures for RNA interference allow large-scale functional screening and provide a promising source of information to systematically identify host factors essential for the replication and propagation of viruses [3-7]. These functional genomics studies have underlined the importance of high-quality "-omics" datasets and the development of integrative and standardized system for their comprehensive analysis, such as the VirHostNet knowledgebase dedicated to virus-host protein-protein interactions [8].

From the late nineteenth-century, Koch's postulates were routinely applied to identify microbial origins of diseases. More recently, molecular postulates were introduced to unravel mechanisms associated to virulence [9]. Indeed, virus-induced diseases originate from the accumulation and combination of perturbations, some of them being driven by direct interactions between viral and cellular components. Therefore, robustness of the virus-infected cell system should correlate to the complex architectural organization and functional regulation of the cellular protein interaction network as well as to the topological nature and intensity of the attack of this network by the virus. We have previously shown that Hepatitis C Virus (HCV) interacts with highly central and bottleneck proteins in the human interactome and built a network model of molecular pathways that are co-deregulated during chronicity [2]. We have also recently compared how viruses interfere with the type I interferon response throughout protein-protein interactions [10]. However systematic associations between viruses and known diseases remain to be explored. In this context Goh et *al*. explored the diseasome network to investigate the molecular basis of genetic diseases throughout integration of the cellular protein interaction network and the comprehensive dataset of disease-gene associations [11,12]. Whether alteration of the host protein network by viruses participate or combine with these genetic predisposition factors to cause complexes diseases is an opened question and has to be investigated at the systems level.

Here, we aimed at providing a systems biology rational to Koch's postulates by deciphering the molecular basis and the complexity of viruses-induced diseases through the comprehensive analysis of virus-host connections to the human diseasome. A bottom-up approach was used to reconstruct the Human Infectome Network (HIN), as a systemic model of virus-infected human cell based on a protein-protein interactions scaffold. The topology of the protein interaction network was first explored and detailed insights on the functional essentiality of host proteins targeted by viruses were given. We next simulated *in silico *topological perturbations associated to viral infection and profiled new candidate targets that could be suitable for drug design. Finally, a prototype of a Human Infectome Diseasome Network (HIDN) based on OMIM annotations of genes involved in human multi-factorial genetic diseases was designed. This framework was applied to explore in more details molecular basis of the Hepatitis C Virus-induced diseases and to question the dual role of viruses in the susceptibility to type 1 *diabetes mellitus *(T1D).

## Results

### Bottom-up reconstruction of the Human Infectome Network (HIN)

**Figure 1:**
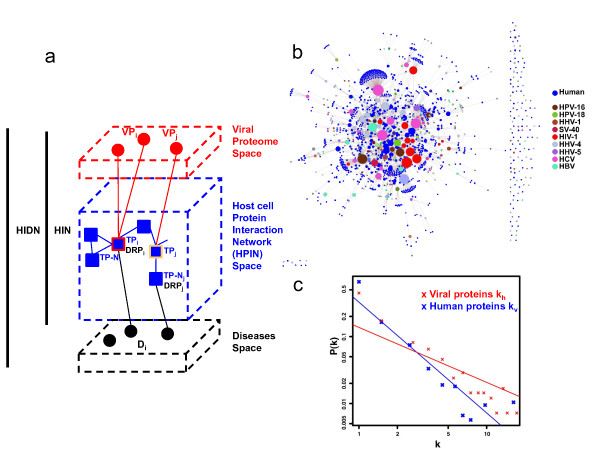
**The Human Infectome Diseasome Newtok**. a. The Human Infectome and Diseasome Network. Viral and host cellular proteins are respectively represented as red and blue nodes. Interactions between host cell proteins are represented by blue edges and define the Host Protein Interaction Network (HPIN). Interactions between viral and host proteins (red edges) are plugged onto the HPIN to define the Human Infectome Network (HIN). Host cell proteins directly interacting with viral proteins are named targeted proteins (TPs). Host cell proteins at a 1-hop distance of a targeted protein are named targeted proteins neighbours - (TP-Ns). Edges between human diseases (black nodes) and host cell proteins represent disease-gene associations as referred by OMIM. Proteins associated to OMIM diseases are termed disease-related proteins (DRPs). DRPs are either directly targeted by viruses (red stroke), or indirectly targeted by viruses (orange stroke). Network integration of HIN with the whole set of disease-gene associations defines the Human Infectome-Diseasome Network (HIDN). b. The virus-host interactome. The virus-host interactome part of HIN is represented as a multi-coloured graph (available in interactive format in Additional file 1). Blue nodes represent host cell proteins and viral proteins are colourized according to their taxonomy origin. Only the most connected viruses among the 110 viruses are represented in the legend: HPV - Human Papillomavirus; SV - Simian virus; HIV - Human Immunodeficiency Virus; HHV - Human Herpes Virus; HCV - Hepatitis C Virus; PTLV - Primate T-lymphotrophic Virus; HBV - Hepatitis B Virus. The size of nodes is proportional to the connectivity of proteins within the virus-host interactome. c. Distribution of viral protein and cellular protein connectivities in the Human Infectome Network. Probability density distributions P(k) of viral protein connectivity (k_h(VP)_) and host protein connectivity values (k_h(TP)_) are respectively given in red and blue and are linearized in *log *scale.

In a systems virology perspective, a virus-infected cell was modelled as an integrated system driven by physical interactions between viral and host cellular proteins - the Human Infectome Network (HIN, Figure 1a). The reconstructed HIN is composed of 416 viral proteins (distributed into 8 viral groups, 28 families and 110 species) that are connected to a reconstructed human interactome (see Methods and Additional file 1) throughout 2,099 manually curated virus-host protein-protein interactions (Figure 1b; full dataset available in Additional file 2 and Additional file 3). Although partial, this viral proteome remarkably interacts with a large panel of host cellular proteins - the targeted proteins (TPs) - representing roughly 5% of the human proteome (n_TP _= 1,148). The probability distribution of the viral targeting intensity, k_v(TP) _- *i.e. *the number of viral proteins interacting with a host protein - showed that 32% (366/1,148) of targeted cellular proteins interact with more than one viral protein. At the taxonomic level, we observed that 28% (316/1,148), 22,5% (259/1,148) and 19,4% (223/1,148) of the targeted cellular proteins interact with more than one virus, family and Baltimore's group respectively. These results are highly suggestive of convergent molecular mechanisms based on protein interactions independently selected during the evolution of viruses.

Approximately 50% of the human proteins (10,378/20,219) have been shown to interact with at least one interacting partner. In our study, a total of 92% (84% with the High Quality HQ dataset; see Additional file 4) of the targeted proteins (n_TP _= 1,055) are also directly and physically interconnected within the human cellular interactome. These results are in good agreement with previous observation made for the Hepatitis C Virus [2]. This large panel of targeted host proteins (Figure 1b) as well as the high connectivity of viral proteins in the cellular network (Figure 1c) - as reflected by the distribution of the number of cellular proteins targeted by a viral protein (k_h(VP)_) - could directly correlate with the functional pleiotropy of viral proteins and with the diversity of molecular strategies they evolved to subvert the host cellular machinery and to evade from the cellular antiviral defence.

### Viruses target highly connected and central host proteins

To better characterize to what extent viral proteins affect the functioning and the robustness of the cellular network, we next analyzed topological properties of targeted proteins within HIN (see Additional file 1 for accurate description of topological properties). As described above, the connectivity (k) of a viral and/or a host protein in the HIN network corresponds to the number of direct interacting partners. The number of host partners of a targeted host protein is denoted k_h(TP)_. The centrality (b) measures the number of shortest paths (spl) (*i.e. *the minimum distance between two proteins in the network, l) that pass through a given protein. The centrality of a protein is used to estimate the theoretical flux of information that is potentially controlled by this protein. We also used the shortest path length observed between human proteins as an unbiased measure of functional relationships as observed within molecular pathways or complexes of proteins.

**Figure 2:**
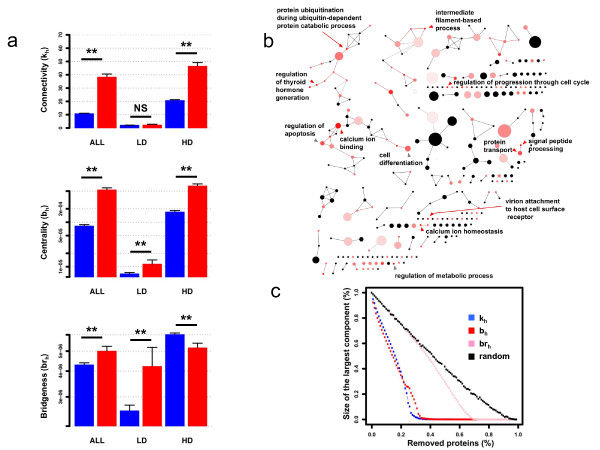
**Topological properties of the Human Infectome Network **. a. Topological properties of virus-targeted proteins. The average connectivity (top), centrality (middle) and bridging centrality (bottom) properties of targeted proteins (red bars) are compared to that of HPIN proteins not targeted by any virus (blue bars). Average measures are split into low connectivity proteins (LD - with a connectivity inferior or equal to 5, the median threshold) and high-connectivity proteins (HD - with connectivity superior to 5). Differences were statistically assessed using one-tailed Wilcoxon test; *P-Values *associated to the test are given (NS - Non significant testing *P-Value *> 0.05; *P-Value *< 0.05 *; *P-Value *< 0.01 **). b. The modular landscape of the Human Infectome Network. The deconvolution of HIN using the CFinder algorithm identified interconnected modules of proteins (nodes) and modules' linkers (available in an interactive format in Additional file 1). Protein modules and linkers are coloured according to the intensity of the viral attack. Highly targeted modules or linkers are red. Poorly targeted modules or linkers are black. Biological processes and molecular functions associated to highly targeted modules are pinpointed (one-tailed Exact Fisher test; Benjamini and Hochberg multiple testing correction; *P-Value *< 0.05 red arrows - *P-Value *< 0.15 grey arrows). c. Simulation of network robustness against preferential viral attack on central and bridging proteins. The figure represents the fragmentation of the entire human protein interaction network (HPIN) according to random or preferential attack according to network properties. The fragmentation is obtained by computing the relative size of the largest connected component (S) as a function of the percentage of removed nodes (f). Nodes removal is performed either randomly (black) or as a preferential attack mode where protein nodes are eliminated from the network in decreasing order of their connectivity (blue), centrality (red) and bridging (pink) values.

Figure 2A shows that viral proteins preferentially interact with highly connected proteins (mean k_h(TP) _= 38 versus mean k_h(NTP) _= 10; one-tailed Wilcoxon test; *P-value *< 2 × 10^-16^; see Additional file 5 for the full distributions), central proteins (mean b_h(TP) _= 5.34 × 10^-04 ^versus mean b_h(NTP) _= 8.23 × 10^-05^; one-tailed Wilcoxon test; *P-value *< 2 × 10^-16^; see Additional file 5 for the full distributions) and closely inter-connected cellular proteins (data not shown, mean spl_h(TP) _= 2.91 versus mean spl_h(NTP) _= 3.66; one-tailed Wilcoxon test; *P-value *< 2 × 10^-16^). These statistically significant trends validate, at a larger scale, previous analysis of experimental virus-host protein interaction networks [1,2]. We next applied a cross-validation procedure based on a high-quality protein-protein interactions dataset (see Additional file 3) to control the potential effect of false positive protein-protein interactions [13]. All the observed trends remained significant, highlighting the robustness of our analysis against false positive bias. This study also shows that cellular proteins targeted by multiple viruses present a distribution shift toward highly connected and central position within the cellular network as compared to proteins targeted by only one viral protein (mean degree = 57 versus mean degree = 29, one-tailed Wilcoxon test; *P-value *< 2.2 × 0^-16^). A similar shift in the distribution was observed at the taxonomic level for viral species (mean degree 62 versus 28), family (mean degree 66 versus 29) and Baltimore's group (mean degree 68 versus 30), confirming previous observations [14]. Although we cannot totally rule out the impact of potential experimental bias (*i.e. *inspection bias due to Y2H screening technology or false positives) until the availability of comprehensive, totally unbiased and high-quality human and virus-host interactomes, it remains that this network-based analysis provides additional support to the hypothesis that viruses have evolved convergent strategies measurable at the proteome-wide level to control highly connected and central proteins in order to subvert essential functions of the cell.

### Deconvolution of HPIN reveals "hot-spots" modules

The host cellular machinery is controlled by a highly complex, dynamic and regulated modular protein interaction network [15]. To understand how the viral proteome interplays with these functional protein modules, we first performed a topological deconvolution of the human protein interaction network into a network of inter-connected modules. The CFinder [16] clustering algorithm was applied to HIN and identified a network of 311 inter-connected modules comprising 6,152 distinct cellular proteins (Figure 2b; Materials and Methods). Gene Ontology functional enrichment analysis showed that most of the characterized topological modules is assigned unambiguously to at least one cellular function (Additional file 6), including molecular transport, cytoskeleton, metabolic pathways, apoptosis, cell cycle and cellular differentiation. Analysis of this modular framework in the specific context of a viral infection showed that 38% of the modules (n = 118) are targeted by at least one viral protein, with 17 highly targeted host-spot modules (red), while other modules are poorly targeted (pink) (Figure 2b; one-tailed Exact Fisher test; Benjamini and Hochberg multiple testing correction; *P-Value *< 0.05). The overlap of proteins observed between modules was finally used to define modules linkers (see Materials and Methods). Interestingly, a statistically significant proportion of these linkers (~20% = 104/541) are targeted by at least one viral protein, suggesting that viruses have a tendency to perturb functional cross-talks between functional modules (simulation with n = 10.000 re-sampling; *P-Value *= 3.0 10^e-4^).

### Targeting bottleneck proteins, an apparent stealth-attack of viruses

We observe a significant correlation between the level of connectivity of host targeted proteins (k_h(TP)_) and the intensity of viral targeting (k_v(TP)_). Nevertheless, the strength of this correlation remains week (r^2 ^= 0.095, P-value < 2.0 × 10^-06^). Although we cannot totally rule out that this partially results from virus-host and host-host interactome incompleteness, it is also highly suggestive of viral strategy based on targeting of poorly connected proteins within the human interactome.

Indeed, the majority of human proteins are poorly connected with 50% of them interacting with only one cellular protein (Figure 1c) even when considering the high-quality HPIN. Most of these poorly connected human proteins are also characterized by heterogeneous measures of centrality within the human interactome as already reported [14]. Hence, following previous observations made on the Hepatitis C Virus protein infection network [2], we next focused on bottleneck properties of targeted proteins defined by their bridging centrality (br) (see Methods).

Interestingly, when focused on the low-connectivity proteins, the analysis revealed that viral proteins significantly interact with bridging proteins, *i.e. *proteins characterized by a higher-level of bridging centrality as compared to the human proteome counterpart (Figure 2a). Because no statistical differences between connectivity of the targeted proteins and the human proteome counterpart is observed at low-connectivity level, we can reasonably conclude that this trend is statistically independent of connectivity properties (top), emphasizing the role of bridging properties *per se*. Using the high-quality dataset, targeting of bridging bottlenecks remains statistically significant, showing the robustness of the data against false positive detection bias (Additional file 1, data not shown). We hypothesized that this category of proteins, by bridging highly connected parts of the cellular network with a limited number of binding events, could be critical checkpoints for the control and regulation of the cellular information flow. In good agreement with this hypothesis, proteins spanning distinct modules of proteins (*i.e. *overlapping proteins) exhibit significant higher bridging centrality than non-overlapping proteins (one-tailed Wilcoxon test; *P-Value *< 2.2 10^e-16^). Taken together, these results support the notion that viruses have evolved highly tuned mechanisms to control host cell functioning. This control can be achieved not only by directly targeting central modular functions, but also by controlling how these functions are co-regulated by targeting molecular bridges.

To further estimate the functional impact of virus-host protein interactions on host cell functions, we next measured topological robustness of the cellular network in a theoretical and simplified model of preferential attack on highly connected, central or bridging proteins. *In silico *simulation of molecular perturbations engendered by protein removal showed that the cellular network exhibits stronger robustness against deletion of bridging proteins as compared to hubs and central proteins removal (Figure 2c, Materials and Methods). Therefore viral targeting of molecular bridges is likely to have a weak impact on the cellular network architecture, minimizing collateral drawbacks on global cellular functioning. However, whether viral targeting of bridging proteins modulates the dynamic and the regulation of essential cellular functions and participates to complex human diseases onset needs further investigation.

### Functional essentiality of targeted proteins for the viruses

**Figure 3:**
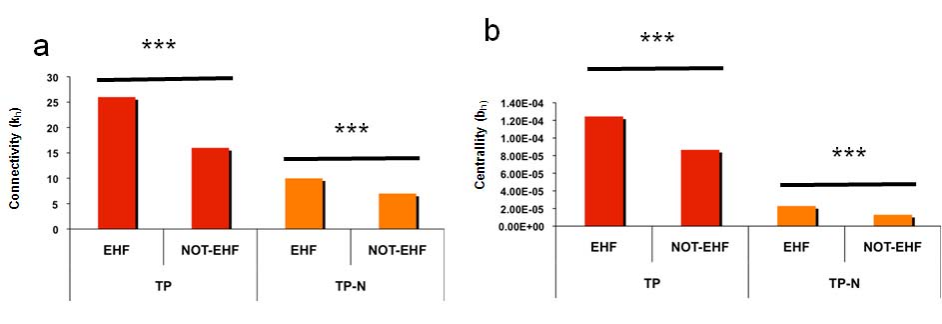
**Cellular connectivity (k_h_) and centrality (b_h_) of Essential Host Factors (EHFs)**. The histograms show the average cellular connectivity (left - k_h_) and centrality (right - b_h_) of Essential Host Factors (EHFs) computed for the group of proteins directly interacting with viruses (left; red bars - TP) and for the groups of proteins indirectly targeted by viruses, (right; orange bars - TP-N). Values are compared to average values of connectivity and centrality computed for a control dataset of not-Essential Host Factors (NOT-EHFs). Distribution differences are statistically assessed using one-tailed Wilcoxon test (*P-Values *< 0.05 *).

To address whether targeted proteins are essential host factors for viral infection, six recent functional RNA interference genomic screens - designed to identify host factors controlling retroviruses and single stranded RNA viral infectivity and replication - were gathered and mapped onto the Human Infectome Network [3-7,17]. A total of 1,501 essential host factors (EHFs) are listed in Additional file 7. The rational of this integrative approach relies on the potential existence of pan-viral host co-factors [6], as a result of shared viral lifecycle steps - including virus integration, replication and egress from the cell - and similar cellular defence mechanisms subverted by viruses. We observe that 10% (164/1,501) of the EHFs are directly targeted by viral proteins as compared to the reference human proteome (odds ratio = 2.38 ranging between 1.98 and 2.83 at 95% confidence interval; one-tailed exact Fisher test; *P-value *< 2.2 10^e-16^). Based on the "guilty by association concept" [18], direct neighbourhood of targeted proteins (TP-Ns) was also investigated to identify indirect interference of viruses with essential host factors. Strikingly, 41.7% of EHFs (626/1,501) were found significantly over-represented within TP-N as compared to the reference human proteome (odds ratio = 1.459 ranging between 1.30 and 1.62 at 95% confidence interval; one-tailed exact Fisher test *P-Value *< 6.427 10^e-12^). Topological analysis also revealed that these essential host factors targeted either directly or indirectly by viruses exhibit a significant higher level of connectivity (one-tailed Wilcoxon test *P-value *< 3.5 10^e-5^) and centrality (one-tailed Wilcoxon test; *P-value *< 4 10^e-^^*2*^) but similar values of bridging properties, as compared to not-essential host factors (NOT-EHFs), *i.e. *proteins that interact with at least one viral protein, but that was not identified as an EHFs by RNA genomic screens (Figure 3). Taken together, these results suggest that preferential targeting of highly connected and central proteins either directly or indirectly correlate, at least in part, with their essentiality for the viral lifecycle.

### Virus targeted proteins and Diseasome

**Figure 4:**
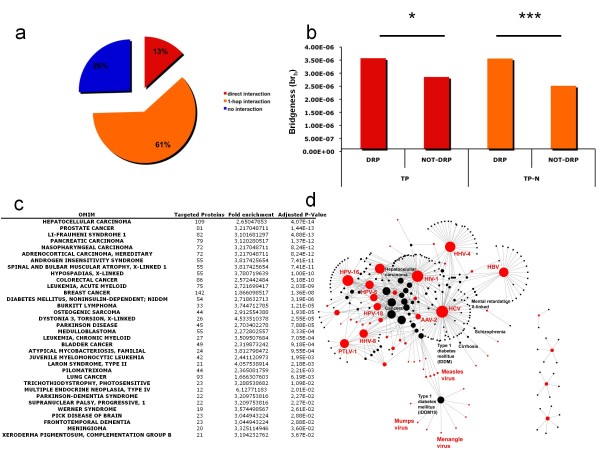
**The Human Infectome-Diseasome Network (HIDN) **. a. Diseasome classification of Targeted Proteins. Viruses interact directly with Disease-Related Proteins (DRPs) but also indirectly throughout 1-hop interaction. The distribution of directly targeted DRPs (TPs, red) or indirectly targeted DRPs (TP-Ns, orange) is given. b. Bridging properties of Disease-Related Proteins. The histograms show average cellular bridging properties (br_h_) of DRPs for proteins directly targeted by viruses (left; red bars - TPs) and for proteins indirectly targeted by viruses, at a 1-hop distance, (right; blue bars - TP-Ns). These average values are compared to average values of bridging computed for a control dataset of not-diseases related proteins (NOT-DRPs). Differences are statistically assessed using one-tailed Wilcoxon test (*P-Value *< 0.05 *; *P-Value *< 0.001 ***). c. OMIM enrichment analysis of the proteins targeted directly and indirectly by viruses. The table shows 34 OMIM diseases significantly connected to viruses both directly and indirectly after multiple testing corrections. For each significant disease, OMIM id, description, disease type, number of targeted proteins, fold enrichment value and the Benjaminin and Hochberg adjusted *P-Value *are given. d. The Human Infectome-Diseasome Network (HIDN). The HIDN was mathematically formalized as a bipartite graph composed of two types of nodes corresponding to either diseases (black nodes) or viruses (coloured nodes). HIDN is composed of 57 viruses and 230 diseases connected by 466 virus-disease associations. In HIDN, disease and viral nodes are connected by an edge if at least one protein related to this disease is targeted by at least one protein encoded by this virus. Viral species and disease are connected by an edge if at least one disease related protein associated to that disease is directly targeted by a protein encoded by the virus. The nodes are sized proportionally to disease connectivity (k_d_) or virus species connectivity (k_vs_) in HIDN.

To decipher the molecular basis of viral infection pathophysiology, the OMIM annotations [19] of 1,729 human genetic diseases related proteins (DRPs) were downloaded (http://www.ncbi.nlm.nih.gov/omim; data are available in Additional file 8) and layered onto HIN (Figure 1a). This integration process showed that 13% (152/1,148) of targeted proteins are significantly directly associated to a least one disease (Figure 4a; Exact Fisher test; *P-Value *< 1.42 10^e-10^). This means that a human protein that directly interacts with a viral protein has roughly 2 times more chance to be involved in a human disease than human proteins not targeted by a virus (odds ratio = 1.86 ranging between 1.54 and 2.22 at a 95% percent confidence interval). This proportion increased to 74,5% (856/1,148) (Figure 4a; Exact Fisher test *P-Value *< 2.2 10^e-16^) when neighbourhood targeting was integrated in the analysis. This strongly suggests that targeted proteins and diseases are tightly connected at the molecular level either directly and/or indirectly. Conversely, 50% (1,007/1,908) of the diseases reported in OMIM were also found connected either directly or indirectly to at least one virus. Statistical enrichment analysis using OMIM ontology identified 34 diseases significantly linked to viruses, the majority of them being related to cancers (lung cancer, hepatocellular carcinoma, breast cancer, leukaemia...) and neurodegenerative diseases (Figure 4c; one-tailed exact Fisher test; Benjamini and Hochberg multiple testing correction; *P-Value *< 0.05). Interestingly, we also showed that the number of diseases associated to TP-Ns is partially correlated to the targeting intensity of these proteins by viral families (r^2 ^= 0.06869; *P-Value=*1.196 10^e-12^).

Network properties of proteins targeted directly or through their neighbourhood were also assessed in the context of the human diseasome. Surprisingly, DRPs directly and indirectly targeted exhibit significant higher bridging values than proteins not connected to any diseases (Figure 4b; one-tailed Wilcoxon test; *P-Value *< 0.05). Interestingly, we have also shown that targeted DRPs exhibit significant higher bridging centrality as compared to not-targeted DRPs (one-tailed Wilcoxon test; *P-Value *< 0.05). This indicates that the viral attack on molecular bridges might participate at least in part to the molecular onset of human diseases.

### Networking the Human Infectome and Diseasome

**Figure 5:**
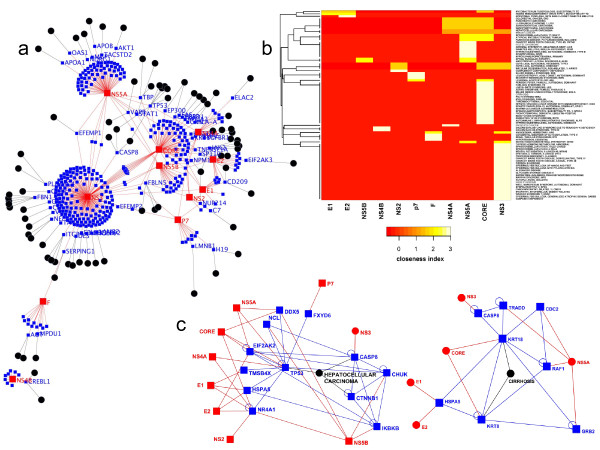
**The Hepatitis C Virus Infectome Diseasome Network**. a. The Hepatitis C Virus Infectome-Diseasome Network. The network is represented as a multi-coloured graph with three types of node: viral proteins (red circle), host cellular proteins (blue circle) and diseases (black circles). Virus-host protein-protein interactions and disease-gene associations are respectively represented by red and black edges. b. Hierarchical clustering of Hepatitis C Virus proteins according to their connectivity to human diseases. The closeness index, *i.e. *the reciprocal of the average distance between viral proteins and diseases were computed within neighbourhood-based HIDN, was used as a distance metric for unsupervised hierarchical clustering. c. HIDN connectivity of HCV proteins and main HCV-associated diseases: Hepatocellular carcinoma (left) and Cirrhosis (right).

According to the apparent tight linkage between viruses and diseases, a prototype of a Human Infectome-Diseasome Network (HIDN) was designed (Figure 4d; see Materials and Methods; Figure 1a). In good agreement with OMIM enrichment analysis, cancers are central to this network and appear mainly connected with DNA viruses (Papillomavirus, Herpes virus) with the noticeable exception of the Hepatitis C Virus, which is associated with chronic infection and chronic diseases. The HIDN model was next applied to explore, without *a priori*, the molecular basis of diseases associated to HCV infection. To take into account potential indirect associations between HCV proteome and DRPs, proteins targeted through their neighbourhood were also included in the analysis (Figure 5a). In a model of molecular perturbations triggered by virus-host protein interactions, we hypothesized that this neighbourhood could to some extent participate to virus-induced diseases. Unsupervised hierarchical clustering of this HCV extended HIDN helped us to profile aetiological relationships between HCV proteins and some well-described HCV-related clinical syndromes (Figure 5b), such as hepatocellular carcinoma (NS4A, NS5A, CORE, NS3) (Figure 5c, left), cirrhosis (CORE) (Figure 5c, right) and to automatically classify and highlight molecular determinants of the diseases. In good agreement with the clustering of CORE and hepatocellular carcinoma, the role of HCV CORE in this disease was previously reported in transgenic mice [20].

**Figure 6:**
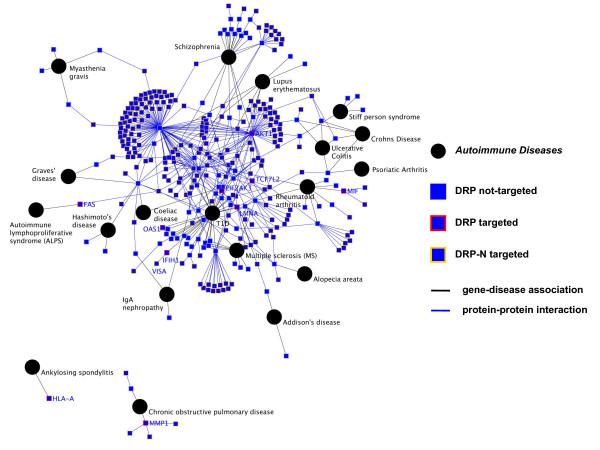
**The Infectome-Autoimmune Diseasome Network**. The Infectome-Autoimmune Diseasome Network is modelled as a multi-coloured graph with two types of nodes (diseases - black circles and host cellular proteins - blue square). Host cellular proteins can be either directly connected to the disease (DRPs) or indirectly connected through 1-hop distance (DRP-Ns). Protein-protein interactions between host cellular proteins are represented by blue edges. Disease-gene associations are represented by black edges. DRPs targeted by viruses are represented with red stroke colour (IFIH1, OAS1). DRP-Ns targeted by viruses are represented with orange stroke colour (VISA).

The extended HIDN was next applied to decipher the molecular aetiology of autoimmune diseases for which viral infection is suspected to play an important role in interaction with genetic factors [21] (Figure 6). A large number of autoimmune diseases catalogued from OMIM also appeared connected to viruses within HIDN (Figure 6). Among them is the type 1 diabetes mellitus (T1D) that exhibits a central position. Whether T1D - an autoimmune disease affecting islet beta cells of the pancreas producing insulin - has a viral origin is still a debate [22,23]. Based on the extended HIDN, we first highlighted a direct connection between different members of the Paramyxoviruses family and T1D. This connection is mediated by an interaction between the viral V protein and the host interferon-induced helicase (IFIH1). Based on the extended HIDN, we also extracted 38 proteins that are targeted by viruses and directly connected to T1D. These proteins could be yet undiscovered predisposition factors that might contribute to the multi-factorial aetiology of T1D. In good agreement with this hypothesis, this set of 38 targeted candidates appeared significantly enriched in functions associated to immunity that could be perturbed during an autoimmune event (for instance proteins involved in innate immune response, cytokine production, lymphocyte T cell differentiation and apoptosis; one-tailed exact Fisher test; Benjamini and Hochberg multiple testing correction; *P-Value *< 1.0 10^e-04^) [24]. Moreover, the mapping of T1DBase expression dataset and QTL information onto T1D HIDN confirmed respectively that 100% of the highlighted candidates are expressed in beta cells and that 50% (18/38) of them are localized within genetically identified T1D susceptibility regions either in human, rat and mouse [25-27] (Additional file 9). Among, these putative genetic factors is the intermediary protein VISA, which is localized in both mouse and rat susceptibility regions.

## Discussion

The Human Infectome Diseasome Network provides a state-of-the-art gateway model towards systems virology. Although the virus-host interactome and the diseasome search space is clearly far from being totally explored, the systems-level analysis proposed here covers more than hundred viruses and thousand of human diseases. The compilation of data summarized 30 years of intensive research on virus-human protein-protein interactions and gene-disease associations. All virus-host protein-protein interactions were manually inspected, guarantying that this resource is not prone to errors of annotation and is strictly composed of physical protein-protein interactions. Careful annotation of the interactions according to PSI-MI standards helped us to address the overall quality of our resource with more than 30% of the interactions (673/2,099) supported by at least two-independent experimental procedures. For the remaining 70% interactions, most of them come from large Y2H screens for which validation rates are closed to 80%, indicating the overall high-quality of our dataset and its robustness for meta-analysis study. This open resource can thus be used as a gold standard for the analysis of pathogen-host interactome but also for the investigation of viral aetiology of human diseases. This resource is available both throughout the VirHostNet website (http://pbildb1.univ-lyon1.fr/virhostnet) and as a supplemental table (Additional file 2).

In depth analysis of well annotated cellular pathways and functions associated to targeted host proteins has previously shown a clear enrichment of cellular functions triggered by the cell in response to pathogens such as innate and adaptive immune response and apoptosis, but also shed light on cellular functions not thought to be associated with viral infection or virus-induced disease [8,14]. Here, the network topology of HIN was intensively studied without any other *prior *knowledge than protein-protein interaction and then was confronted to independent functional genomics and genetics data.

First of all, by projecting RNA interference functional genomics data onto HIN, we observed that viruses have a strong tendency to directly and indirectly target proteins that are essential for viral infectivity and replication and that these proteins are characterized by high values of connectivity and centrality in the cellular network. These cellular "hubs" may appear as potential drug targets in the pharmaceutical quest of viral replication inhibitors. However, because of the functional centrality of these targets and their large impact on protein network architecture and robustness we can anticipate that drug designed on this rational could generate molecules with high toxicity. As an emergent topological property of HIN, we also highlighted a massive attack of viruses on bridging proteins. As suggested by the analysis of modules extracted with the CFinder algorithm, these bridging proteins may participate to cross-talks between defined functions and contribute to the plasticity of the whole cellular system [28]. Because of the difficulties of partitioning highly connected networks, the improvement of clustering algorithms and/or network pre-treatments procedures should be considered in further studies to accurately delineate those functional modules and bridging proteins connecting them in a more specific context, for instance by integrating transcriptomic and/or proteomic changes induced by viral infection. As suggested by recent reports and our data, these bridging proteins could be key targets for drug development [29,30]. Indeed, we showed that perturbations associated to molecular bridges disruption have a lower impact on network topology than highly connected and central proteins. It should be noted however that integrative analysis of RNAi data did not revealed specific bridging properties for EHF. Any conclusion drawn from available RNAi experiments and virus-host interactome must be taken cautiously regarding the relative small number of viruses for which functional and physical mapping were performed. However, because the cellular network is robust to the targeting of bridging proteins it can reasonably be assumed that these proteins express their essentiality in a combinatory way rather than in isolation. Interestingly our analysis of directly or indirectly targeted bridges revealed that these proteins could play a role in predisposition to some diseases. Such significant proportion of essential host factors indirectly targeted by viruses is really promising in the specific context of antiviral drug discovery focusing on host proteins, as viral adaptation against these "stealth targets" is unlikely and will not favour appearance of drug resistance. Altogether, our results suggest that multiple targeting of bridges could be an innovative network-based drug-discovery strategy to increase the anti-viral arsenal with low toxicity molecules.

Comprehensive combination of virus-host interactome with other "-omics" data (*i.e. *regulatory maps, functional genomics screens) and genome-wide association studies will allow deciphering of virus-host complex relationship as well as a better understanding of the role of viruses in the aetiology of some complex multi-factorial human diseases. The simplified model that is proposed here will have to be improved for instance by measuring how interactions between viral and host proteins are essentials for the virus or yet dispensable for the life of the host. It will be also of great interest to better formalise and quantify how do the viral proteins interact with the cellular proteins to completely abrogate the function of the host proteins and how often do they modulate their functions by hijacking the cellular proteins for their own needs.

The Human Infectome and Diseasome Network framework also helped us to revisit and complete Koch's postulates, initially developed to characterize microbial causation of infectious diseases, with the fresh eye of molecular systems virology [31]. Indeed, by taking into account molecular connectivity between viruses and diseases, the Human Infectome-Diseasome Network generates new mechanistic hypothesis concerning their molecular relationships. Even if partially biased towards best-studied viruses or proteins, a comprehensive analysis of HIDN shows that these infectious agents, such as HCV, are central to a wide range of pathologies, most of them being related to cancers. This observed association between viruses and cancers, although statistically highly significant, remains low most likely because the OMIM database annotates almost exclusively inherited diseases linked to germinal mutations. Association would likely be much stronger if diseases linked to somatic mutations were included in the study. It is also important to note that the actual data cannot accurately distinguish between the causative and preventive role of virus-host protein-protein interactions in the onset of human diseases, which would require additional *in vitro *and *in vivo *validation analysis. However, our results suggest that accumulation of molecular perturbations triggered throughout virus-host protein-protein interactions could participate to the aetiology of diseases along with genetic or environmental predisposition. This may be the case for the IFIH1 protein targeted by Paramyxoviruses. Interestingly, IFIH1 gene has recently been identified by two independent genome-wide association studies as a susceptibility locus for T1D [32-34]. IFIH1 is a cytoplasmic helicase triggering interferon antiviral signal in response to viral RNA exposure. Nejentsev et *al. *hypothesized that frequent variants of IFIH1 are selected to efficiently fight viral infection whereas rare variants disrupting the native IFIH1 function may protect from T1D risk. It is noteworthy that V-IFIH1 protein-protein interaction inhibits production of Interferon-β, a cytokine directly involved in the pathogenesis of T1D [35]. Interestingly, it has been suggested that a viral infection could abrogate an ongoing autoimmune destruction of pancreatic cells, mainly through immuno-modulatory mechanisms [36]. One non-restrictive scenario would be that modulation of IFIH1 by Paramyxoviruses participates to this event. Another major protein of innate immunity is directly targeted by HCV and connected to T1D via NS5A-OAS1 protein-protein interaction [37,38]. Based on the extended HIDN framework, we also explored the direct protein partners of known T1D-related proteins that are directly targeted by viruses. In this context we introduce the "*pris la main dans le sac*" ("caught red-handed") concept, a new concept to pinpoint the functional importance of those proteins targeted by pathogens that will undoubtedly provide a rational for prioritizing new molecular susceptibility factors. Among the 38 "*pris la main dans le sac*" candidates indirectly interacting with disease related proteins, VISA is known to be involved in the production of IFN-β in an IFIH1-dependent pathway and could be crucial role in T1D susceptibility. Although too preliminary to draw general conclusions on the viral aetiology of autoimmunity, these observations nevertheless support the idea that a comprehensive analysis of virus-host protein-protein interactions and their immuno-modulatory consequences at the systems level is a powerful approach for the identification of susceptibility factors and new therapeutic strategies [39,40].

## Conclusions

By merging the Human Infectome and Diseasome Networks, we provided the first bottom-up systems-level mapping of molecular associations between viral infection and human diseases based on virus-host physical protein-protein interactions. This association is consistent with the hypothesis that the onset and the pathogenesis of some chronic diseases, even mainly attributed to genetics, lifestyle and environment factors may also be modulated by infectious agents. The Human Infectome Diseasome Networks also provide an original platform and resource to rationally identify therapeutic targets.

## Methods

### Coloured graph modelling of the Human Infectome Network (HIN)

The Human Infectome Network (HIN) was modelled as a coloured graph. A coloured graph is a graph which has colours associated with each type of vertex or each type of edge. HIN is composed of two coloured vertex types - viral (V_v_; red) and host proteins (V_h_; blue) and two coloured edges types - virus-host (E_vh_; red) and host-host (E_hh_; blue) protein-protein interactions (Figure 1a). HIN was formalized as a multi-coloured graph G_HIN _= (V, E); with a finite series of graph vertices V defined in {V_h_, V_v_} and a set of graph edges E defined in {E_vh_, E_hh_}. An integrative bottom-up strategy was used to reconstruct a high-quality prototype of HIN based on experimental protein-protein interactions data manually extracted from the literature and integrated from different public databases. Virus-host protein-protein interactions rely on a high-quality dataset manually curated and validated by a team of virology and molecular biology experts, as described in VirHostNet [8] (Additional file 2). This highly reliable virus-host protein interactome was then plugged onto a human protein interactome reconstructed from 9 public databases (see Additional file 1 for more details on the reconstruction process, Additional file 3). The whole HIN resource is publicly and interactively available within the VirHostNet knowledgebase (http://pbildb1.univ-lyon1.fr/virhostnet).

### Community clustering of HIN

The CFinder (http://cfinder.org) percolation algorithm was performed on the cellular protein interaction network part of HIN to construct a network of interconnected cellular proteins' modules. More details on the methodology are available in Palla *et al. *[16]. We used the fourth k-clique level to define modules. In this network, each node corresponds to a module of interconnected proteins and each edge corresponds to the set of overlapping proteins connecting modules (the modules linkers). This network is composed of 311 modules, including 1 giant component corresponding to highly central proteins and 310 modules in periphery. Gene Ontology annotation (http://www.geneontology.org) was used to characterise the functional relevance of each module using functional enrichment approach based on exact Fisher testing and Benjamini and Hochberg multiple testing correction. The top biological process with significant enrichment (Benjamini and Hochberg correction; *P-Value *< 0.05) for more than half of the corresponding proteins was retained to attribute a function to a given module (Additional file 6). Virus-host protein-protein interactions were next projected onto this network to quantify the level to which modules and linkers are targeted. In this context, a permutation test based on *n *= 10,000 re-sampling was performed to test the significance of a viral attack on linkers. The *P-value *was then estimated as the proportion of re-sampled values as, or more, extreme than the observed value.

### The bridging centrality measurement

To quantitatively characterize bridging properties at the systems-level, bridging centrality measurement derived from Hwang et Ramanathan [41], was computed for each protein of the human cellular network.

The bridging centrality br(v) for node v of interest, is defined by:

br(v)=b(v)×bc(v)

The bridging coefficient is defined by

$$\text{bc}(\text{v})=\frac{1}{\text{k}(\text{v})}\times \sum_{\text{i}\in \text{N}(\text{v})}\frac{1}{\text{k}(\text{i})}$$

where N(v) is the set of neighbours of the node v.

### In silico simulation of network topology perturbations

We implemented the methodology developed by Jeong et *al. *to systematically quantify and compare the extent of network perturbations associated to nodes removal, in a simplified model of viral attack. Decrease of the largest component size was monitored as a function of the proportion of nodes removed and was compared for the different topological metrics (degree, betweeness, bridging centrality) [42].

### Coloured graph modelling of the Human Infectome-Diseaseome Network (HIDN)

The HIDN was mathematically formalized as a bipartite graph composed of two types of nodes corresponding to either diseases (black nodes) or viruses (coloured nodes). In HIDN, disease and viral nodes are connected by an edge if at least one protein related to a given disease is targeted by at least one protein encoded by this virus. Viral species and disease are connected by an edge if at least one disease related protein is directly targeted by at least one viral protein. The extended HIDN is a collapsed protein-protein representation of HIDN that includes the direct neighbours of the disease-related proteins that are targeted by viruses.

### Statistical analysis

The Wilcoxon rank-sum test was used to statistically test for the differences between medians of variables not-normally distributed. The Wilcoxon rank-sum or Mann-Whitney U test is a non-parametric test which is used as an alternative to the two-sample Student's t-test, for assessing whether two independent samples of observations come from the same distribution. The Exact Fisher test was used to statistically test the differences observed between proportions and to test overrepresentation and functional enrichment. Benjamini and Hochberg correction was used to adjust *P-value *associated to multiple testing (adjusted *P-Values *< 0.05 were considered as significant). A total of 20,219 non-redundant coding genes were used as the human proteome reference dataset for all the statistical enrichment tests.

## Abbreviations

DRPs: Disease Related Proteins; DRP-Ns: Disease Related Proteins Neighbourhoods; EHFs: Essential Host Factors; EHF-Ns: Essential Host Factors Neighbourhoods; HIN: Human Infectome Network; HIDN: Human Infectome-Diseasome Network; N-DRPs: Not Disease Related Proteins; N-EHFs: Not Essential Host Factors; N-TPs: Not Targeted Proteins; T1D: Type 1 Diabetes mellitus; TPs: Targeted Proteins; TP-N s: Targeted Proteins Neighbourhoods

## Authors' contributions

VN designed, performed the study and drafted the manuscript. BDC was involved in data analysis. BDC and VL and CRC corrected the manuscript. All authors read and approved the final manuscript.

## Supplementary Material

Additional file 1 **Supplementary material**. This file contains the supplementary material and data of the manuscript. Click here for file

Additional file 2 **Virus-host protein-protein interactions**. The dataset of 2,099 virus-host protein-protein interactions. Click here for file

Additional file 3 **Human protein-protein interactions (HPIN)**. The full and high-quality datasets of respectively 70,874 human and 36,144 human protein-protein interactions (HPIN). Click here for file

Additional file 4 **Network properties**. Network properties with the full and the high-quality Human Protein Interaction Network. Click here for file

Additional file 5 **Connectivity and centrality probability distribution of targeted proteins (TP) within the human interactome part of HIN**. P(k_h_) is the probability of a node to connect k_h _other nodes in the network. P(b_h_) is the probability of a node to have a centrality equal to b_h _in the network. Normalised *log *degree (top) and *log *centrality (bottom) distribution of not-targeted (blue) and targeted proteins (red). Solid lines represent linear regression fits. Vertical dashed lines give mean degree and centrality values. Each class is represented with conventional standard error. Click here for file

Additional file 6 **The modular landscape of the Human Infectome Network. Virus-Host targeting profiles and Functional annotations**. Each line represents a module of proteins (or a community) extracted from the HIN using CFinder algorithm. Each community is identified by a unique identification number (*community_id*) that is also used to describe the module in the SF1.tar.gz interactive network (HIN_modules_landscape_network.properties, see Additional file 1). The total number (*n_tot*) of proteins forming the module, the number (*n_inf*) and proportion (*prop_inf*) of proteins targeted by viral proteins as well as the Exact Fisher test P-value (*P-value*) of the preferential targeting of the module are given. GO functional enrichment of each module has been computed and the gene ontology accession number (*go_acc*), its description (*description*), the proportion of protein in this module having the selected go term (see main text Methods) (go_prop_annot), the proportion of proteins of the modules being annotated by at least one term (go_n_annot) are given for both biological process and molecular function annotation. Click here for file

Additional file 7 **Human Essential Host Factors (EHFs)**. The full dataset of 1,501 human Essential Host Factors (EHFs). Click here for file

Additional file 8 **Diseases Related Proteins (DRPs)**. The full dataset of Diseases Related Proteins and their associations with genetic diseases described in OMIM. Click here for file

Additional file 9 **T1DBase annotation of the 38 T1D candidates**. Genomic Co-localization of the candidates with a susceptibility region to T1D (*Genetic Confirmed Region*) in either human, mouse or rat; expression in beta cells/Islets (*Expression in Beta Cells/Islets*) and manual curation by T1DBase (*T1D Annotation*) are annotated by Boolean ("1"). The number of publications with T1D candidate gene co-citation is also given (*T1D Publications*). Click here for file
