# Supplementary material for: When the human viral infectome and diseasome networks collide: towards a systems biology platform for the aetiology of human diseases
Source: BMC Syst Biol. 2011 Jan 21;5:13. doi: 10.1186/1752-0509-5-13 (PMC3037315; doi:10.1186/1752-0509-5-13)
Supplement: Additional file 1 — Supplementary material. This file contains the supplementary material and data of the manuscript. [file 1752-0509-5-13-S1.DOC]

**When the human viral infectome and diseasome networks collide: towards a systems biology platform for the aetiology of human diseases**

Vincent Navratil ^1,2,$^, Benoit de Chassey ^1,3^, Chantal Rabourdin Combe ^1,3^, Vincent Lotteau ^1,3,4^

^1^ Université de Lyon, IFR128 BioSciences Lyon-Gerland, Lyon 69007, France

^2^ INRA, UMR754, rétrovirus et pathologie comparée, Lyon 69007, France

^3^ Inserm Unit 851, Lyon 69007, France

^4^ Hospices Civils de Lyon, Hôpital de la Croix-Rousse, Laboratoire de virologie, Lyon 69004, France

^$^ Current address: Pôle Rhône Alpes de Bioinformatique, Université Lyon 1, Batiment Gregor Mendel, 16 rue Raphaël Dubois, 69622 Villeurbanne cedex, France

**Email addresses:**

[navratil@prabi.fr](mailto:vincent.navratil@univ-lyon1.fr)

[benoit.dechassey@inserm.fr](mailto:benoit.dechassey@inserm.fr)

[chantal.rabourdin@inserm.fr](mailto:chantal.rabourdin@inserm.fr)

[vincent.lotteau@inserm.fr](mailto:vincent.lotteau@inserm.fr)

**Corresponding authors:** [navratil@prabi.fr](mailto:vincent.navratil@univ-lyon1.fr)

# Additional file 1

## Reconstruction of a Human Protein Interaction Network (HPIN).

An integrated *Human Protein Interaction Network* (HPIN) was reconstructed from 9 public databases - including BIND, Intact, MINT, HPRD, DIP, Generif, Biogrid, REACTOME, Networkin - as previously described in *Navratil et al.* [1]. In a first step, protein accession numbers and official gene names associated to each partner were mapped and unified onto Ensembl protein accession numbers (ENSP#). Then PSI:MI controlled vocabulary and Pubmed identification numbers (PMID) were retrieved from the database to annotate each protein-protein interaction (ppi). Altogether, the reconstructed HPIN is composed of 70,874 non-redundant and unique ppis involving more than 10,000 human protein partners. As previously recommended by Cusick et al. [2], to prevent false positive bias, only high-confidence ppis supported by at least two different experimental procedures or two independent PMIDs were subsequently retained from the full HPIN. The resulting high-quality (HQ) HPIN is composed of 36,144 ppis involving 7,917 human proteins and was used as a control dataset to cross-validate all significant trends identified from the full HPIN. Both full and HQ HPIN datasets are available in Additional file 3.

## Topological metrics of HIN and HIDN networks

Distinct network metrics, based on graph theory, were extended to the HIN multi-coloured graph:

**- connectivity (*k) -*** The degree or connectivity k of a vertex v in a graph G summarises the number of edges that are incident to this node. The degree is a local centrality measure as it takes into account only direct 1-hop neighbourhood, *i.e.* the direct interacting partners.

**- betweenness or centrality *(b)* -** The centrality b of a vertex v in a graph G can be defined roughly by the number of shortest paths going through a node v. This value is normalised by roughly twice the total number of protein pairs in the graph (n*(n-1)). The equation used to compute centrality, b(v), for a node v is:


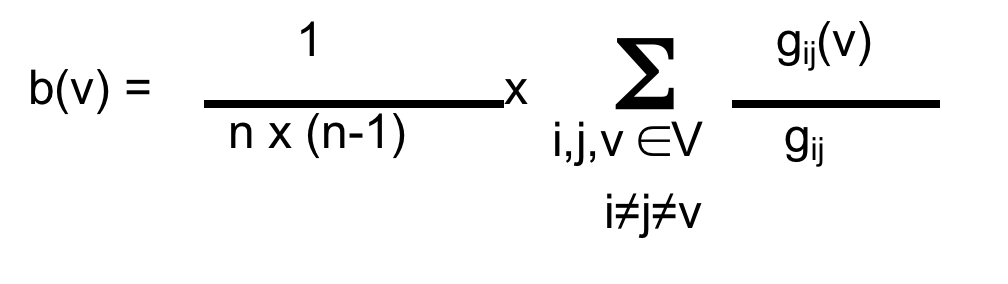


where g_ij_ is the number of shortest paths going from node i to j, i and j ∈ V and g_ij_(v) the number of shortest paths from i to j that pass through the node v.

b(v) =

n x (n-1)

x

**Σ**

g_ij_(v)

g_ij_

i≠j≠v

1

The median value of the degree metric was used to define without *a priori* a threshold for the definition of low degree (LD) and high-degree (HD) proteins within HIN. This was also used to compare network characteristics of both targeted and not-targeted LD and HD proteins.

**- bridging centrality (*br)* -** We have previously shown that cellular degree (k_h_) and betweenness (b_h_) measures of host proteins are partially correlated within the human cellular network [3]. We have also demonstrated, when considering low degree proteins (LD), that Hepatitis C Virus proteins tend to preferentially interact with highly central ones, *i.e.* the bottleneck or bridging proteins. Indeed, LD proteins might exhibit higher bottlenecks properties than HD, which intrinsically exhibits more alternative paths in a similar range of betweenness values.

To quantitatively characterize bridging properties at the systems-level, bridging centrality measurement derived from Hwang et Ramanathan work [4], was computed for each protein of the human cellular network.

The bridging centrality br(v) for node v of interest, is defined by:

br(v) = b(v) × bc (v)

The bridging coefficient is defined by


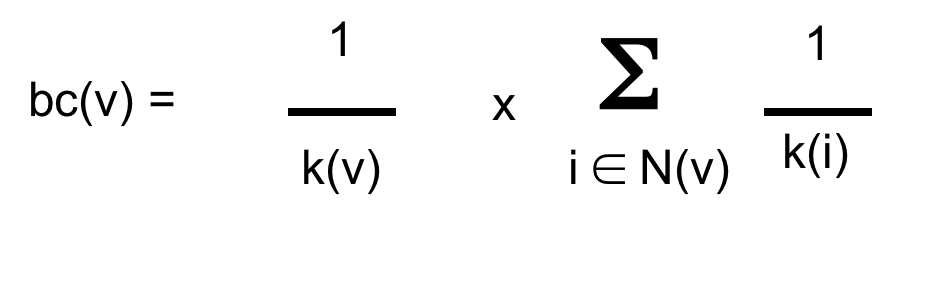


where N(v) is the set of neighbours of node v.

The median value of the bridging centrality metrics was used to define a threshold for the definition of bridging proteins (BPs) and not-bridging proteins (N-BPs).

To check the robustness of our bridging bottleneck measurement against false positive detection bias, bridging centrality was computed in both the Full and HQ HPIN and was found significantly correlated (Spearman’s rank correlation coefficient *ρ*=0.53, P-Value < 2.2 10^e-16^).

**Topological metrics within HIN multi-coloured graph.**

The definition of connectivity, centrality, bridgeness metrics was specialized, according to the multiplicity of vertices and edges types within the coloured graph HIN:

**- k_h_**, the host connectivity of a protein, *i.e.* the number of host proteins interacting with this protein

**- k_v_**, the viral connectivity of a vertex*, i.e.* the number of viral proteins interacting with the HIN protein

**- b_h_**, the host centrality of a vertex within the human interactome part of HIN

**- br_h_**, the host bridgeness of a vertex within the human interactome part of HIN

**Topological metrics within HIDN bipartite graph.**

The definition of connectivity was specialized, according to the multiplicity of vertices within the bipartite HIDN graph.

**- k_d_**, the disease connectivity associated to a virus, *i.e.* the number of disease connected to a virus.

**- k_vs_**, the viral species connectivity of a disease*, i.e.* the number of viral species connected to a disease.

Within HIDN, (Figure 4a) the nodes are sized proportionally to either disease connectivity (k_d_) or virus species connectivity (k_vs_) in HIDN.

## Network metrics computation

The R ([http://www.r-project.org](http://www.r-project.org/)) statistical environment was used to perform statistical analysis and the igraph R package (<http://cneurocvs.rmki.kfki.hu/igraph>) to compute network centrality measures (deree, betweenness) and implement bridging centrality.

## HIN and HIDN networks visualisation

Guess tool ([http://graphexploration.cond.org](http://graphexploration.cond.org/)) was used to graphically represent HIN, the modular landscape of HIN and HIDN (full view, Hepatitis C Virus and type 1 diabetes mellitus centred). Figure 1a, Figure 2a, Figure 4d, Figure 5a, Figure 6 are also given in a GUESS interactive format (Figure 1a: HIN_network.properties, Figure 2a: HIN_modules_landscape_network.properties, Figure 4d: HIDN_network.properties, Figure 5a: HCV_network.properties and Figure 6: AI_network.properties) available at <http://vinavratil.free.fr/navratil_hidn>. Full instructions are given in the README.pdf file.

## Functional genomics screening data integration

Essential host factors (EHFs) for the viral life cycle were integrated from 6 recent functional genomics screens [5-10]. EHFs are characterized by measures of centrality ranging between the average centrality computed for the cellular network (**Wilcoxon test *P-value* = 1.332e-15)** and the highest centrality of proteins targeted by viruses (**Wilcoxon test *P-value*= 3.2 e-16).**

# References

1. Navratil V, de Chassey B, Meyniel L, Delmotte S, Gautier C, Andre P, Lotteau V, Rabourdin-Combe C: **VirHostNet: a knowledge base for the management and the analysis of proteome-wide virus-host interaction networks.** *Nucleic Acids Res* 2009, **37:**D661-668.

2. Cusick ME, Yu H, Smolyar A, Venkatesan K, Carvunis AR, Simonis N, Rual JF, Borick H, Braun P, Dreze M, et al: **Literature-curated protein interaction datasets.** *Nat Methods* 2009, **6:**39-46.

3. de Chassey B, Navratil V, Tafforeau L, Hiet MS, Aublin-Gex A, Agaugue S, Meiffren G, Pradezynski F, Faria BF, Chantier T, et al: **Hepatitis C virus infection protein network.** *Mol Syst Biol* 2008, **4:**230.

4. Hwang WC, Y.R. C, A. Z, Ramanathan M: **Bridging Centrality: Identiffying Briding Nodes In Scale-free Networks.** *KDD'06 August 20-23, Philadelphia, PA, USA* 2006.

5. Hao L, Sakurai A, Watanabe T, Sorensen E, Nidom CA, Newton MA, Ahlquist P, Kawaoka Y: **Drosophila RNAi screen identifies host genes important for influenza virus replication.** *Nature* 2008, **454:**890-893.

6. Krishnan MN, Ng A, Sukumaran B, Gilfoy FD, Uchil PD, Sultana H, Brass AL, Adametz R, Tsui M, Qian F, et al: **RNA interference screen for human genes associated with West Nile virus infection.** *Nature* 2008, **455:**242-245.

7. Konig R, Zhou Y, Elleder D, Diamond TL, Bonamy GM, Irelan JT, Chiang CY, Tu BP, De Jesus PD, Lilley CE, et al: **Global analysis of host-pathogen interactions that regulate early-stage HIV-1 replication.** *Cell* 2008, **135:**49-60.

8. Zhou H, Xu M, Huang Q, Gates AT, Zhang XD, Castle JC, Stec E, Ferrer M, Strulovici B, Hazuda DJ, Espeseth AS: **Genome-scale RNAi screen for host factors required for HIV replication.** *Cell Host Microbe* 2008, **4:**495-504.

9. Brass AL, Dykxhoorn DM, Benita Y, Yan N, Engelman A, Xavier RJ, Lieberman J, Elledge SJ: **Identification of host proteins required for HIV infection through a functional genomic screen.** *Science* 2008, **319:**921-926.

10. Tai AW, Benita Y, Peng LF, Kim SS, Sakamoto N, Xavier RJ, Chung RT: **A functional genomic screen identifies cellular cofactors of hepatitis C virus replication.** *Cell Host Microbe* 2009, **5:**298-307.
