# Supplementary figures and images for: When the human viral infectome and diseasome networks collide: towards a systems biology platform for the aetiology of human diseases
Source: BMC Syst Biol. 2011 Jan 21;5:13. doi: 10.1186/1752-0509-5-13 (PMC3037315; doi:10.1186/1752-0509-5-13)

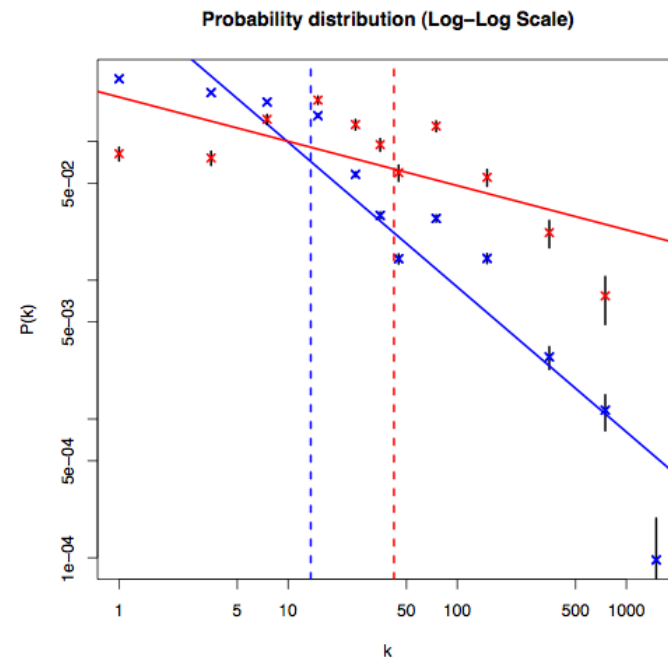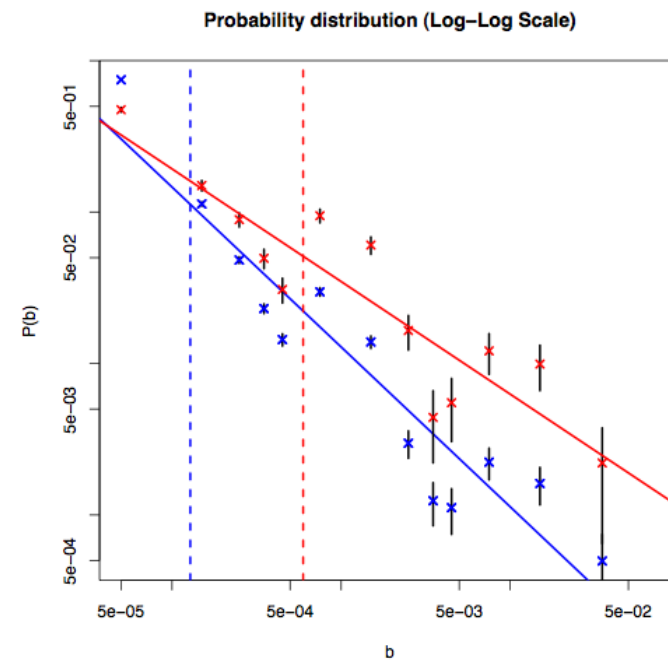

Supplement: Additional file 5 — Connectivity and centrality probability distribution of targeted proteins (TP) within the human interactome part of HIN. P(kh) is the probability of a node to connect kh other nodes in the network. P(bh) is the probability of a node to have a centrality equal to bh in the network. Normalised log degree (top) and log centrality (bottom) distribution of not-targeted (blue) and targeted proteins (red). Solid lines represent linear regression fits. Vertical dashed lines give mean degree and centrality values. Each class is represented with conventional standard error. [file 1752-0509-5-13-S5.PDF]
